# Type and efficacy of online training for informal carers: a narrative review

**DOI:** 10.3389/fpubh.2025.1603020

**Published:** 2025-05-30

**Authors:** D. Bressington, R. Gray, K. Barclay, C. Overy, I. Blackberry

**Affiliations:** ^1^Care Economy Research Institute, La Trobe University, Albury-Wodonga, VIC, Australia; ^2^Faculty of Nursing, Chiang Mai University, Chiang Mai, Thailand; ^3^School of Nursing and Midwifery, La Trobe University, Bundoora, VIC, Australia; ^4^La Trobe Rural Health School, La Trobe University, Albury-Wodonga, VIC, Australia; ^5^John Richards Centre for Rural Ageing Research, La Trobe Rural Health School, La Trobe University, Albury-Wodonga, VIC, Australia

**Keywords:** eLearning, online training, informal carers, caregivers, older people, dementia, support

## Abstract

**Background:**

The need to provide training and support for unpaid informal carers of people with various health conditions is well established. This narrative literature review was conducted to explore recent evidence of informal carer eLearning education and training programs, and to guide the design of an online training program for Australian carers, including those living in rural and geographically remote settings.

**Method:**

Different combinations of relevant search terms were used across three databases: Google Scholar Advanced, MEDLINE with full text incorporating PUBMED, and CINAHL to search for literature published since 2014. Articles within peer-reviewed journals were chosen based on their topic relevance and strength of evidence.

**Results:**

The recent systematic reviews highlight that there is a lack of good quality research evidence for the range and efficacy of eLearning programs designed for carers of veterans and adults with disabilities, or mental health conditions, with most evidence related to carers of older people and people with dementia.

**Conclusion:**

Online educational programs for carers of older people and/or older people with dementia, and those with mental health conditions can improve carer well-being. Future programs should be co-designed with carers, evaluate care recipient outcomes, and address recruitment, retention, and information technology skills.

## Introduction

This narrative literature review was conducted to guide the design of an effective asynchronous online informal carer eLearning program for Australian carers, including those living in rural or geographically remote settings.

Informal carers have been defined as people who provide unpaid health and social care for someone in the context of an existing relationship (i.e., usually family members, neighbors or friends) (1). Informal carers make an essential contribution to the global care and support economy; for example, estimates suggest that approximately 2.65 million Australians provide informal care annually (2), amounting to a cost equivalent of around $77 billion (3).

Informal carers (for the purposes of this article, will hereafter also be referred to as carers or caregivers) have been shown to experience a reduction in health status and quality of life as a result of caring (4, 5). They often overlook their own needs due to prioritizing the care recipient (6). Carers residing in rural and remote geographical regions may experience particularly high levels of mental health concerns due to increased care workload, problems accessing formal support services and social isolation (7). The poor well-being of informal carers has been reported to be exacerbated by the social restrictions and changes in formal care service delivery during the COVID-19 pandemic (8–10).

The impacts on carers that were highlighted due to the COVID-19 epidemic have in turn highlighted the importance of providing training and support to carers. Psychoeducational interventions may ameliorate caregiving burden (11) and the need to provide training and support to improve caregiving skills for carers of people with a variety of health conditions is well established (12). Online programs, including eLearning and mobile health applications (apps) can afford greater program accessibility and learning flexibility (13). The extant literature shows that various eLearning approaches have promise for different groups of carers (14). This includes those who provide care for important care recipient subgroups, such as older people, and older people with dementia (15), military veterans (16), adults with disabilities (17) and people with mental health difficulties (18). For this review we focused on the above areas of care provision, as they were also identified by the National Taskforce in the Australian Government Draft National Care and Support Economy Strategy (19), and we included mental health due to an Institute research specialization.

It is important to explore existing studies and design new fully online-based programs as such programs can incorporate innovative technologies like artificial intelligence (AI) and virtual reality (VR) to promote learning. The eLearning format is also more accessible for people living in rural areas and unable to reach distant face-to-face training and support programs. In addition, online learning can be conducted asynchronously, which means that the learning can be accessed at any time that suits the carer. This is especially advantageous for carers who are balancing their own employment while providing care.

Several systematic reviews and primary research studies have been published in each area of informal care provision, some combine eLearning with other non-online training interventions, including complex psychosocial support and psychoeducational interventions facilitated by professionals and peers [i.e., (12, 20)]. However, there have been no recently published narrative reviews of the efficacy of informal carer eLearning programs that synthesize the extent of evidence arising from studies conducted with different care recipient groups. Given the lack of synthesized evidence, it is difficult to confidently establish the effects of such programs, complicating the design of new and effective online approaches to educate and support carers. Thus, this review aimed to extrapolate research findings relevant to the review question and narratively synthesize the evidence of efficacy to identify the nature and outcomes of eLearning programs for carers reported in selected studies. It also aimed to explore the nature and efficacy of informal carer eLearning programs on community-dwelling carer and care recipient outcomes in four areas of care: older people and/or older people with dementia, veterans, mental ill-health and disability.

## Methods

This is a narrative review of recent evidence of the efficacy of eLearning programs for informal carers. Articles reporting studies of *eLearning education/training programs* were considered for selection to include in this review if they clearly labelled as such, or were identified by the authors as approaches to support carers and their care recipients through learning new information, skills or coping strategies via online learning. Single studies which utilized live (synchronous) online sessions or those that were delivered face-to-face, were not selected for review (unless they were within an included systematic review) because the main objective of this literature review was to inform the design of a subsequent asynchronous e-learning program. Some individual studies using a blended learning approach were selected for inclusion where there was limited evidence on wholly asynchronous online approaches and the results provided useful information to answer the review question.

### Study selection

Full-text articles published in English within peer-reviewed journals were chosen for inclusion and narrative synthesis based on their topic relevance and strength of evidence as indicated by where they are situated in the Evans (21) hierarchy of evidence for effectiveness (i.e., anecdotal evidence and expert opinion the lowest and systematic reviews with meta-analysis the highest). Included articles were also selected based on their recency, with articles published in the last 5 years prioritized.

Therefore, recent relevant systematic reviews (or other types of reviews if no systematic reviews available) were selected first to answer the review question. In the absence of a recent review, or if additional experimental studies had been published since the publication of a review, the relevant recent primary intervention studies were included for narrative review. Studies with broader topic relevance were selected for inclusion where there were insufficient directly relevant primary studies and some of the findings provided useful insights.

### Search terms

Different combinations of relevant search terms (varied for each database) were used across three databases (Google Scholar Advanced, MEDLINE with full text incorporating PUBMED, CINAHL) to search for literature published since 2014. The reference lists of selected articles and their citations in Google Scholar were also searched. The searching was an iterative process. Different combinations of search terms and Boolean operators were used to focus results and filter out irrelevant articles as necessary (for example using “NOT dementia” when trying to identify studies involving older people without cognitive impairment). Example search terms used within MEDLINE were:

1st level: online learning OR e-learning OR distance learning OR remote learning OR virtual learning OR online education.
2nd level: informal caregivers OR family caregivers OR informal carers OR family carers OR carers OR caregivers OR relatives.
3rd level (varied terms to determine area of care): mental health OR mental illness OR mental disorder OR psychiatric illness OR mental well-being; older people OR older adults OR elderly OR dementia OR Alzheimer’s OR cognitive impairment OR memory loss OR dementia patient OR people with dementia; veterans OR military OR soldiers OR servicemen; disability OR disabilities OR disabled.

## Results

The Google Scholar Advanced search was limited to 200 results, whereas results for other databases yielded up to 400 articles depending on variations in the use of search terms, Boolean operators and other filters (i.e., adults aged 19 + years). The overall extent of the literature and study outcomes for each area of care recipients are discussed below.

### Online training for carers of older people and/or people with dementia

A great deal of literature was identified reporting studies of the effects of eLearning and/or supportive online psychoeducational interventions for carers of people with dementia and to a lesser degree, older people more generally.

The large extent of the literature is reflected by a review of systematic reviews having been published in the last few years regarding caring for older people more broadly. This recent umbrella review of systematic reviews synthesizes the results of previous reviews of interventions designed to mitigate the negative health outcomes of informal caregiving to older adults (12). The review included 47 systematic reviews of quantitative and qualitative studies, encompassing a wide variety of complex interventions delivered across a range of formats for different long-term physical and neurological conditions, including dementia.

The Kirvalidze et al. (12) umbrella review conducted in 2023 identified 19 earlier reviews focused on both psychosocial and educational interventions for carers of older people; however, only one (15) was focused on online delivery of educational and supportive interventions for carers of people with dementia. This review did not calculate pooled effect sizes using meta-analyses due to significant levels of heterogeneity and reported that the overall quality of included studies was low. The Hopwood (15) review included in the umbrella review tentatively reports that the interventions may be beneficial for carers’ well-being (i.e., depression, care burden and anxiety) and that the provision of online information was most effective if it was part of a multicomponent intervention and tailored for individual carers. Overall, Kirvalidze’s et al. (12) umbrella review concludes that no more systematic reviews are required on this topic because the existing reviews all include some poor-quality primary intervention studies. Instead, more robust controlled trials of discrete eLearning programs are needed. The review also highlighted that the extant evidence of effectiveness was inconclusive due to highly discordant quantitative results.

In relation to caring for people with dementia specifically, the most directly relevant and methodologically sound systematic review identified was entitled: “Remotely delivered information, training and support for informal caregivers of people with dementia” and was conducted by researchers associated with the Cochrane Collaboration (11). This systematic review and meta-analysis included 26 randomized controlled trials (RCTs) published up to April 2020. Most of the studied interventions (23 of 26) included the provision of information as a component of a more complex supportive psychoeducational intervention. Only two studies were included that reported pure training interventions (22, 23), and therefore, it is impossible to draw firm conclusions about the pooled effects of such programs. Conflicting somewhat with the findings of some earlier less robust systematic reviews [i.e., (15)], this well-conducted systematic review and meta-analysis (11) highlighted that remotely delivered information, training and support interventions for caregivers of people with dementia result in no statistically significant reductions in caregiver burden, caregiver knowledge/skills and caregiver quality of life, and there were very small improvements in depressive symptoms (SMD = −0.25) when compared to the provision of information alone. It was also observed that the carers allocated to the complex intervention groups experienced higher rates of attrition than those in the information alone control groups (pooled risk ratio of 1.51). None of the included studies measured outcomes of the care recipients. Taken together, the findings of this Cochrane review reveal that adding supportive psychosocial interventions to eLearning programs for carers of people with dementia (i.e., telephone-delivered or online facilitated peer or professional support) may result in slightly better outcomes, but risks increasing drop-out rates, although it is unclear why this is so.

A slightly updated and more focused systematic review and meta-analysis entitled: “The effectiveness of internet-based psychoeducation programs for caregivers of people living with dementia: a systematic review and meta-analysis” (24) was published after the above-mentioned Cochrane review and umbrella review of reviews. The review included non-randomized experimental studies, unlike the Cochrane review (11). This methodologically robust review also comprehensively explored the components of the eLearning programs and included a total of 19 articles published over the last 30 years, 13 of which were pooled in the meta-analysis. The strength of evidence was presented in accordance with GRADE [Grades of Recommendation, Assessment, Development, and Evaluation—a systematic approach to rating the certainty of evidence; (25)]. The review concluded that most of the 13 included RCTs had a low risk of bias, whereas four RCTs were assessed as having some concern of bias relating to randomization and allocation concealment (24).

Similarly to the more broadly-focused Cochrane review (11) of RCTs, Yu’s meta-analysis revealed that the reviewed online psychoeducational programs only had a small significant effect of reduction on caregivers’ depressive symptoms (SMD = −0.19), a small-moderate significant effect on reduction of caregivers’ stress (SMD = 0.29), but no effects on caregiver burden, quality of life, anxiety or self-efficacy. No care recipient outcomes were reported or analyzed in the review (24).

In terms of program characteristics in Yu’s review of internet-based interventions, all psychoeducational programs included components of self-directed learning and provision for text-based internet functions (i.e., information, discussion boards and email). Ten of the included programs did not have functionality for active synchronous learning or support, whereas seven programs included opportunities to interact with a health care professional/facilitator via different modes, including telephone, social media group chat, videoconferencing and online discussion boards (24). The programs with peer-support components also tended to use similar communication channels, with an increasing use of social media observed in the newer studies.

Overall, the Yu et al. (24) review identified several aspects of program content that contributed towards the positive outcomes, including addressing *carers’ factors* and *care recipient factors*. Important carers’ factors to address included building carers’ identity and positive views of the relationship with the person with dementia and how to be a caregiver in the context of existing relationships with the care recipient (i.e., a spouse-caregiver or caring for a parent). Content that seemed effective to address care recipient factors, included the prevention and management of changes in behavior associated with dementia, how to address functional deficits and how to provide practical care, such as activities of daily living (24).

In terms of individual studies of eLearning programs for carers of people with dementia, arguably the most popular and commonly studied program seems to be the dementia iSupport program. This online training and skills program was developed by the World Health Organization (WHO) to improve the quality of life of caregivers (26). The program incorporates problem-solving and cognitive behavioral approaches to provide education, skills training, and support for carers. It comprises five modules: (i) introduction to dementia; (ii) being a caregiver; (iii) caring for me; (iv) providing everyday care; and (v) dealing with behavior changes. A review of clinical trial protocol records revealed that the program has been adapted at least 31 times for different settings and has been translated into 27 languages (27). Published evaluations of iSupport were included in the previously mentioned systematic review (24) however, currently, there is no discrete systematic review and meta-analysis of iSupport studies, therefore its pooled effect size remains unknown.

Regarding individual iSupport studies, a recent pilot RCT of the Portuguese version (26) including 42 participants, reported that there were no significant differences between iSupport and the e-book comparison group in caregiver burden, depression, anxiety, quality of life, positive aspects of caregiving, and self-efficacy when using intention-to-treat age-adjusted analyses. However, the retention rate was only 52.4% in the iSupport group and when per-protocol analysis was conducted (i.e., using only data from participants completing the iSupport group) significant group-by-time interaction effects favoring the iSupport group were observed in carers’ levels of anxiety and environmental quality of life. The positive per-protocol analysis results highlight the importance of the relationship between good attendance and effectiveness and suggests more emphasis should be placed on engaging and retaining participants in the program to realize its potential. Conversely, the study retention rate for the e-book control group was 95.2%, perhaps indicating that the control intervention had good acceptability. Despite the relatively low engagement with iSupport, the findings from the qualitative study data highlight that most carers endorsed the program as a good source of support, reminders to engage with the program were helpful, they were satisfied with the contents, design and functionalities, and they were motivated to participate as they hoped to get information about management strategies of the disease and to obtain emotional support. The drawbacks of the program were that less educated, or digitally illiterate carers, felt excluded and there was a lack of face-to-face personal interaction (26).

The Portuguese iSupport results concur with an earlier RCT of the Indian version (28), which included 55 carers and reported that there were no significant differences between the control and intervention groups in depression, care burden, self-efficacy, mastery and self-rated health. Only one secondary outcome, which was caregivers’ person-centered attitude, was observed to have significantly improved at 3-months follow-up, with poor study retention rates of 36% also reported.

Results of other studies which have adapted and are currently evaluating iSupport programs for the Netherlands (29), the United Kingdom (UK) (30) and Chinese-Australian caregivers (31, 32) have not, at the time of this writing, yet published their full evaluation results, therefore it remains unclear if these versions of iSupport will achieve better results than the earlier studies or experience similar intervention adherence and retention rate issues. A study protocol for an international multi-site trial of iSupport has been published, with an additional ‘Virtual Assistant’ component [iSupport VA; (33)] in Australia, Indonesia, New Zealand, and Vietnam. This adapted program aims to improve the previously noted suboptimal iSupport attendance and engagement rates by making it more user-friendly and by including an accompanying app that helps the user identify solutions for caregiving problems in real time. According to the trial registration details, this multi-site study is ongoing, and results have yet to be published.

The recent published work in the area also seems to highlight that there is a tendency to neglect examining care recipient outcomes, therefore it is impossible to confidently ascertain if modest improvements in carers’ mental well-being translate into better outcomes for older care recipients with and without dementia. However, a systematic review (34) of non-online psychosocial interventions for dyads of dementia carers and care recipients provides some indication of what effects may be expected on care recipient outcomes if these were delivered online. The review reports that the meta-analysis showed no statistically significant pooled effects despite 14 of the 22 included RCTs reporting positive outcomes (34). Of note, the most effective interventions were delivered over the medium term, three to four months, and were observed to involve educating the carer on dementia and appropriate communication skills.

A broader systematic review of training interventions for caregivers and their older care recipients (35) reports similarly mixed results to Balvert’s et al. (34) review, concluding that although the extent of evidence is limited, the interventions can be effective to reduce caregivers’ stress and may also result in a subsequent improvement in older care recipients’ quality of life. However, the Aksoydan et al. (35) review included only two studies that delivered the intervention entirely using an eLearning platform (36, 37), and both studies did not evaluate care recipient outcomes. An RCT of a face-to-face educational program on Alzheimer’s disease patients’ quality of life (38) which was not included in Aksoydan’s (35) review also highlights that care recipients’ self-reported quality of life can improve significantly when carer and care recipient dyads are engaged in dementia educational programs in person rather than online. These findings highlight the need to conduct additional good quality primary studies of eLearning programs to ascertain if similarly positive results can be achieved when programs are delivered totally online.

A focus group study appraising online resources for older people and frail adults also offers some insights into the views of carers about the nature and contents of online programs in five European countries Papa et al. (3). Analysis of the eight focus groups revealed that carers were keen for carer-focused online resources to be available but were previously unaware of specific sources. Carers cited poor information technology (IT) skills and dubious reliability of sources as the main barriers to accessing resources. They also identified four categories of useful resources: carers’ well-being, managing health and diseases of the care recipient, useful contacts, and technologies for eldercare (39). These categories can be considered as important elements to be included in subsequent eLearning programs for carers.

In summary, despite the large extent of literature on the effects of eLearning programs for informal carers of older people and people with dementia, the strength of evidence for efficacy on carer outcomes is questionable and some conflicting findings in the quantitative outcomes are noted across individual studies. However, it is consistently reported from systematic reviews with meta-analyses and several primary intervention studies that the programs result in small to moderate improvements in caregivers’ levels of depression, and possibly anxiety and stress. The Dementia iSupport is the most evaluated program internationally, however the evidence of its efficacy is also limited, and attendance and adherence rates tend to be quite low. No studies seemed to have evaluated the effects of eLearning programs (delivered solely online) on care recipient outcomes, however educational programs delivered face-to-face or via blended appearances may result in modest improvements in care recipients’ self-reported quality of life.

### Online training for carers of veterans

The extent of literature is more limited for carers of veterans than that observed for older people, or older people with dementia, and people with mental ill-health. We were unable to identify any directly relevant recent systematic reviews and meta- analyses of eLearning programs for carers of veterans. Hence, the extant evidence on the effects of educational programs for this informal carer group originates from limited primary research studies. However, a recent scoping review of computerized health interventions for Australian veterans and their families and two non-systematic reviews exploring broader concepts associated with carers of veterans were found, and although they do not completely answer the review question, these provide some potentially useful information to guide the development of relevant educational programs.

The recent scoping review on computerized health interventions for Australian veterans and their families (40) identified ten studies reporting relevant interventions, three of which were computerized educational interventions. The review appears to be well conducted with a replicable search strategy included. However, only three studies evaluating outcomes were included and these were uncontrolled studies. None of these studies evaluated educational interventions specifically designed for both veterans and their carers. The review concludes that most computerized interventions were focused on the mental health of veterans to help them reintegrate into civilian life. Generally, this scoping review highlights that there is a lack of Australian-focused computerized health educational interventions for family carers of veterans and very few controlled experimental studies to evaluate training intervention outcomes (40).

The second review, a critical narrative review of literature published on informal caregivers’ inclusion in veteran’s care, was published in 2021 (16). The review was conducted using systematic methods (with replicable search strategy) and included 35 relevant papers published between 2005 and 2017, of which 30 reported qualitative studies. The remaining five studies comprised three quantitative studies and two conceptual designs. Unfortunately, this review did not evaluate the quality of included studies, therefore the strength of the evidence is unclear. The analysis and narrative synthesis resulted in identifying five major themes related to the concept of inclusive care: *clear definition of caregiver role*, *system level policies for inclusion*, *explicit involvement of caregiver*, *provider assessment of caregiver capability*, and *mutuality in caregiver–provider communication.* The theme relating to a *clear definition of caregiving role* is of particular relevance to the current review, highlighting carers’ need for information and support for the provision of basic personal care tasks, emotional support, navigating and coordinating care, risk management and advocating for the care recipient. These needs seem especially pertinent for new carers. The Boucher et al. (16) review also outlined four intervention studies designed to improve carer inclusion in veterans’ care, which have some promise, however none of these are educational interventions/training programs delivered exclusively online.

The third identified review (41) entitled “How eLearning Can Decrease Challenges of Informal Family Caregivers of Service Members & Veterans with Invisible Injuries” is a narrative literature review that does not provide adequate details of the review methods or search terms used, nor does it assess the quality of included studies. There was also no attempt to synthesize results, quantify effect sizes from individual studies or calculate the pooled effects of relevant e-learning interventions. Hence, the strength of evidence underpinning the review’s conclusions is questionable. The Goodson (3) review includes a substantial amount of research evidence that is not veteran-specific, and its main focus was on identifying important development issues rather than evaluating the efficacy of eLearning. Despite the methodological shortfalls of the review and lack of included studies on eLearning efficacy, it provides useful general information to guide the subsequent development of eLearning programs. The review concluded that learning modules for carers of veterans with invisible injuries (i.e., mental health related) should address self-care techniques, materials to share with those new to the caregiving role, effective ways to communicate with a medical team, and useful methods of treatment for invisible injuries.

There was also a lack of identified recent primary research studies (i.e., individual RCTs) reporting the efficacy of eLearning programs for informal carers of veterans. The majority of recent evidence originates from a federally mandated veteran carer support program in the United States (US) [the Program of Comprehensive Assistance for Family Caregivers (PCAFC); (42)]. However, this program is facilitated using some blended learning, it includes the provision of financial support, is delivered partially in a face-to-face group format and most evaluations of the program rely solely on qualitative feedback. Therefore, evaluations of this nationwide program are unable to answer the question posed in the current narrative review.

A mixed-methods study by Yank et al. (43) provides some insight into how to maximize engagement with online psychoeducational programs for informal carers of veterans. This study explores major stakeholders’ perspectives on an older web-based psychoeducational and self-management workshop for informal carers of veterans (Building Better Caregivers; BBC) funded by the US Department for Veterans Affairs and implemented nationwide since 2013 (44). The BBC program was implemented nationwide in the US based on its purported effects in reducing depression, pain, stress and caregiver burden for informal carers of veterans at 3-months follow-up (44). However, the study reporting BBC’s effects is of relatively low quality as it was a pilot study with a single group of 60 carers (45) and its subsequent uptake nationwide was lower than expected. The program is also facilitated by professionals in a live online workshop format, rather than being delivered as an asynchronous eLearning program. Yank’s et al. (43) study concludes that the uptake of BBC and similar programs would be improved if potential participants believed the online workshop would result in a positive impact, if there were diverse program delivery options and if there was greater investment in outreach and marketing capabilities, including detailed and personal marketing.

The recent wider literature on studies of informal carers of veterans seems to be largely focused on determining the socio-economic and psychosocial impacts of caring for veterans along with their support needs. For example, Jacobs et al. (46) and Gillin et al. (47) report the care impacts and support requirements of carers of veterans in the US and UK, respectively. These studies clearly convey the care needs and provide some direction to meet these, but they do not test the efficacy of relevant eLearning programs per se.

In summary, although it is clearly established that informal carers of veterans experience significant challenges, thus requiring additional support and education (40, 48), there is a distinct lack of controlled trial evidence for effective eLearning programs to address these issues. The majority of evidence of their efficacy originates from US-based program evaluations that are not delivered solely online and are not well-controlled, hence they are heavily subject to bias.

### Online training for carers of people with mental ill-health conditions

Most of the evidence on the efficacy of informal carer eLearning education and training programs on mental health carer and care recipient outcomes originates from online trials of psychoeducational interventions for carers of people with psychosis. The evolution of these programs over the last 20 years or so stems from the early studies of face-to-face family interventions first conducted in the 1990s (49). These trials highlighted that family therapy when delivered face-to-face with a single family was effective in reducing rates of psychotic relapse, shortening hospital admissions and enhancing antipsychotic treatment adherence (49). Family interventions when delivered in-person have also been shown to improve the well-being of family carers, particularly regarding carer burden, stress levels and reducing family conflict (50). These interventions have become relatively widespread due to their success and because some clinical guidelines suggest that family-based intervention should be provided for people with psychosis and their families to improve both the patients’ and carers’ outcomes [i.e., (51)]. This foundation of evidence and clinical need has subsequently led to some online versions of psychoeducational interventions for families being evaluated over the last 10 years.

Several systematic reviews of psychoeducational interventions have been published over the last few years [i.e., (20, 52)], reporting significant improvements in family caregiving burden and carers’ perceived stress, in addition to fewer symptoms and hospitalizations for the care recipient. However, Okafor’s and Manahan (20) review and meta-analysis includes studies utilizing psychoeducation that were delivered face-to face, either individually or in groups, and is focused on the efficacy of online interventions on carer burden alone, neglecting to analyze other important outcomes. Whereas Barbeito (3) review included feasibility along with other outcomes, concluding that online family interventions are promising because they are well-accepted, with good levels of adherence and satisfaction. However, a lack of good-quality RCTs precludes drawing firm conclusions about their efficacy (52).

A recent systematic review (53) focusing on the effectiveness of internet-based psychosocial interventions among family caregivers of people with schizophrenia on a range of outcomes has superseded Barbeito (3) review. Kaewwanna et al.’s review included five relevant trials published between 2010 and 2022, four of which were RCTs. The online interventions consisted of active online psychoeducation (three studies), passive online psychoeducation (two studies), and one study of a smartphone-based problem-solving self-learning program. However, only two of these included studies were conducted fully online, with the others using a blend of face-to-face and online formats. The review concluded that no firm conclusions can be drawn about efficacy because the included trials report conflicting findings and there was significant methodological heterogeneity precluding meta-analysis (53). The small number of studies conducted solely online also precludes confident estimates of their efficacy when compared to other delivery formats.

Given the lack of directly relevant systematic review evidence, it is important to consider relevant contemporary primary research studies. The most relevant, a fully powered robust RCT, was recently conducted in the UK (18) to evaluate the effects of a digital psychoeducation and peer support program (COPe-support) compared to a ‘passive online information resource’ on the mental well-being of family carers supporting individuals with psychosis. The trial included 407 family carer participants, results showed that there were no statistically significant differences between the two groups on the primary outcome (mental well-being) or any of the secondary outcomes at 10 and 40 week- follow-up. Small non-significant improvements in mental well-being were observed in both groups, however the passive online information group improved more than the COPe-support group. The researchers partially attribute the lack of observed effect to participants’ suboptimal engagement with the COPe-support intervention, resulting in an insufficient intervention dose (18) and potentially an unexpectedly effective control condition. Despite the lack of observed effect, the results of this study are relevant to the subsequent design of an informal carer eLearning program. The study authors concluded that the engagement data indicated digital technology is useful to meet carers’ psychoeducational needs and the format can help to build a network of carer support across large geographical distances (18). Generally, the results also highlight that there is indeed some benefit to carers of people with mental illness engaging with online information resources that do not include aspects of peer-support, as the control condition also experienced some improvements in outcomes.

The majority of earlier family-inclusive psychoeducational programs are designed for care recipients diagnosed with psychoses, therefore evidence of efficacy for carers of people with common mental disorders (i.e., anxiety and/or depression) is lacking. A recent Australian RCT (54) is the exception as it tests the efficacy of a novel online early intervention program (*Minds Together*) for carers of people with depressive or anxiety symptoms. This RCT included 127 Australian carers to evaluate the effects of adding a social support platform to an earlier *Minds Together* psychoeducational online program demonstrated to have good feasibility and acceptability (55). Participants in the new trial were randomly allocated to either the original Minds Together program or the Minds Together program plus an online social forum (54). Both groups received 10 weeks of intervention and outcomes were measured immediately post intervention and at 12-week follow-up. Trial outcomes were carers’ quality of life, perceived social support, care burden, coping self-efficacy and psychological distress. The original Minds Together program comprised four online learning modules released weekly and 6 weeks of unrestricted access to the program. The four modules included: The caring journey; Caring for yourself and others; What matters to you and how to talk about it; and Helpful strategies for everyday life (55). The learning modules aimed to improve carers’ health literacy, develop cognitive behavioral therapy (CBT) related skills to improve the relationship with the care recipient and build coping skills (54). Intention-to-treat analysis with full imputation of missing data revealed no statistically significant differences between the two groups on any outcomes (54). However, when both groups were combined, there was an observed statistically significant improvement in carers’ quality of life at 12 weeks follow-up. Unfortunately, the trial reports poor engagement and follow-up rates, with 56% of Minds Together participants and only 20% of the Minds Together plus social support group completing the programs (54). The low engagement rates undermine confidence in the analysis results and indicate that the addition of the social support group is associated with higher rates of discontinuation than the original program.

Overall, the extant evidence on the effectiveness of eLearning programs for informal carers of people with mental health conditions is somewhat opaque due to most tested interventions adopting complex educational interventions with a range of delivery formats and methods. Although there is good quality evidence suggesting that family-inclusive psychoeducational interventions are effective for carer and care recipient outcomes, it is not clear how well these approaches work when delivered online. The observed positive outcomes in control groups of the most recent RCTs of eLearning/online supportive interventions seem to suggest that there is no benefit in adding additional live peer or social support components to eLearning programs for carers of people with mental health conditions. Although the theoretical benefit of social and peer support is obvious, adding these components seems to present engagement and retention challenges within controlled intervention studies.

### Online training for carers of adults with disabilities

The degree of recent literature focused on eLearning programs for informal carers of adults with disabilities is more limited than any other area of care. Most reviews conducted over the last 10 years tend to include studies involving care recipients with a range of long-term conditions [i.e., (56)] or focus on specific aspects of physical health in people with intellectual disabilities [i.e., dental health—(57)]. Whereas primary research studies often focus on functional disability occurring within the context of multimorbidity, for example, physical disability in people with dementia (17) or predominantly study carers of children with intellectual or developmental disabilities [i.e., (57)].

There appears to be no published systematic review of the efficacy of eLearning programs for informal carers of adults with physical or intellectual disabilities. The only recent relevant systematic review identified (59) starkly illustrates the limited extent of studies conducted with carers of adults with disabilities. This review of international literature aimed to establish the effectiveness of online programs for family carers of people with intellectual disabilities, however only two small feasibility trials were eligible for inclusion in the review, and these were both conducted with carers of children, rather than adults (59).

Concerning primary research studies, no controlled trials were found that specifically evaluate an eLearning program solely designed for informal carers of adults with intellectual or physical disabilities. However, a trial published within the last 5 years (60) reports the effectiveness of a mobile-learning program (HiSense APP-ID) in improving knowledge, empathy, and self-efficacy in professional caregivers of persons with intellectual disabilities (ID). The HiSense APP-ID eLearning program comprises a series of short daily sessions with repeated use of the same multiple-choice questions to promote knowledge retention and recall. The program includes six main topics delivered over 8 weeks: (1) attachment theory in daily practice, (2) socio-emotional functioning in persons with ID, (3) sensitivity and responsiveness to communicative signals, (4) emotion regulation, (5) observation and interpretation of behavior, and (6) basic knowledge about ID and common comorbidities (60). The trial originally intended to recruit both informal and formal carers and was therefore designed with informal carers in mind, however it was unable to recruit any informal carers. Finally, the study included 101 professional carers who were randomly allocated to the intervention (HiSense APP-ID) or control (waitlist) groups. Results showed that theoretical knowledge improved more within the intervention group than the control group at post intervention (8-weeks from baseline) and at follow-up (13-weeks from baseline) when measured using a multiple-choice questionnaire. A statistically significant group-time effect was found at follow-up for empathic concern (favoring the intervention group) however, no significant effects were observed for social empathy or self-efficacy (60). Overall, the study indicates that the HiSense APP-ID is a flexible and useful educational tool that improves knowledge retention and recall, and may enhance empathy in professional carers of people with mild to moderate ID. The efficacy of this program should be considered in light of the biases arising from methodological limitations. All outcome measures were self-completed and repeated throughout the study, hence they are heavily subject to social desirability reporting bias. Similarly, the multiple-choice questionnaire measuring knowledge was repeated throughout the study, which may promote strategic learning and hence can only measure participants’ ability to recall specific course contents.

A recently published Australian mixed-methods evaluation of an online Carer Wellbeing and Connection program (61) is worthy of consideration, despite including informal carers with a mix of intellectual, mental and physical support needs, as the sample consisted of around 40% of carers for people with Autism or an intellectual disability. The uncontrolled study recruited 103 informal carer participants who self-rated psychological distress, social support and loneliness before and after completing the program. The program comprised four live facilitated 90-min small group sessions (morning, afternoon, and evening choices), including contents on carer connection and belonging, carers’ values, confidence building and exploring self-identity. The eLearning was supported by a trained facilitator and technical support advisor and pre-session readings were emailed in advance. The program also offered respite care for the care recipient via Carers Australia. The co-designed 4-week program and follow-up assessment was completed by 83% (86) of recruited carers. The quantitative results showed statistically significant improvements in all outcome measures immediately following the program (Coe at l., 2023). However, these findings need to be considered as being heavily subject to bias as there was no control group and the self-reported measures risk introducing reporting bias. Given the provision of respite care as an addition to the program it is also difficult to be confident in the findings due to the potential confounding effects. All sessions were also facilitated rather than being delivered asynchronously. The qualitative interview data analysis (from interviews with 67 carers) identified eleven ‘ingredients for success’, which may be useful for designing new programs. These were: “1. Delivery by a trained facilitator; 2. Provision of respite for the person being cared for during meetings; 3. Technical assistance; 4. Online modality; 5. Inclusivity; 6. Diversity of experience; 7. Shared understanding; 8. Safety; 9. Emotional release; 10. Reflection, and; 11. Self-care practices” [(61), p. 1].

There is a clear lack of good quality evidence supporting the use of eLearning programs for informal carers of adults with disabilities. The best evidence originates from studies that are heterogenous in nature, include the provision of live support from a facilitator, are uncontrolled evaluations and utilize outcome measures that are subject to bias.

## Discussion

This narrative review aimed to establish the nature and effects of eLearning programs on community-dwelling carer and care recipient outcomes. Several clear research gaps were identified along with useful information on program contents, which can be used to guide the design and research evaluation of new programs.

### Overall extent of literature

The amount of published systematic reviews clearly illustrates how extensively eLearning programs have been evaluated across the different care recipient groups. At least 47 systematic reviews on the effects of eLearning/supportive online psychoeducational interventions for informal carers of older people are available and several recent systematic reviews on carers of people with mental health problems have been published. Whereas very few systematic reviews have focused on informal carers of veterans, and none have been conducted that include studies with informal carers of adults with disabilities. Overall, the recent systematic reviews highlight that there is a lack of good quality research evidence for the efficacy of eLearning programs designed for informal carers of veterans and adults with disabilities, with most robust evidence related to carers of people with dementia. Although a large body of psychoeducation intervention literature has been built over the last 20 years, the evidence on the efficacy of eLearning programs for carers of people with mental health conditions is also relatively limited. This is because most of the existing evidence is related to the provision of face-to-face supportive psychosocial therapeutic interventions, which are delivered online, rather than evaluations of eLearning programs that do not incorporate regular and extensive live supportive sessions (i.e., are primarily asynchronous).

### Efficacy of eLearning on carer and care recipient outcomes

Despite the overall lack of trial evidence, studies using less robust designs typically report that informal online carer educational and supportive interventions are beneficial for carers’ well-being (i.e., depression, stress, care burden and anxiety) and that the provision of online information was most effective if it was part of a multicomponent intervention and tailored for individual carers. There was only limited evidence of the efficacy of wholly online programs. In part, this was because identifying studies of programs delivered solely online was challenging as they were given a variety of labels, including training, eLearning, psychoeducational interventions and supportive psychosocial interventions. However, in reality these overlap significantly as most programs are relatively complex and include elements of support and self-management rather than only providing information and/or a defined program of online learning. These heterogeneous studies were also pooled in some systematic reviews, undermining confident estimates of effects. Therefore, it is uncertain if the positive results reported in some individual studies of more complex interventions can be achieved when programs are delivered totally online. The majority of evidence on the effects of asynchronous eLearning for carers of veterans, people with dementia and people with psychosis originates from control groups, where they were being used to compare their effects with more complex supportive interventions. This trial evidence suggests that adding synchronous professional and peer support and other intervention elements to eLearning programs may result in small to moderate benefits, but this increases the risk of dropouts.

Most primary research evidence supporting the efficacy of informal carer eLearning programs originates from uncontrolled single-group program evaluations or small pilot RCT studies. Although such evaluations can measure within-group improvements and obtain useful qualitative feedback, they are relatively low on the hierarchy of evidence and thus subject to a high degree of bias. Despite this, some programs seem to be widely disseminated based on this relatively weak evidence, including the WHO’s iSupport, which has 31 different versions, but currently has no good quality evidence of effectiveness and the attendance and retention rates are relatively low (i.e., around 30% use the program over the longer term). A large funded international multi-site feasibility RCT of an adapted version of iSupport is also ongoing, which is notable given the current lack of evidence supporting the effects of the existing program. The funding and dissemination of eLearning programs based on questionable evidence is concerning and may be attributed to a range of issues which should be addressed. It was observed that many studies were small-scale feasibility pilot evaluations that are likely to have received very limited funding, which may be insufficient for robust evaluations such as RCTs. This situation may also arise due to funding bodies and institutions investing in programs without insisting on a robust evaluation of effectiveness. It is also possible that the nature of eLearning programs does not lend itself well to controlled evaluations, such as randomized controlled trials due to challenges measuring outcomes objectively.

There are several other potential reasons for the lack of trial evidence and sometimes conflicting trial outcomes, which should be considered when designing new studies/programs. Some well-designed controlled studies [i.e., (60)] experienced challenges recruiting informal carers, while many others [i.e., (18, 26, 54)] reported poor attendance and retention rates. These practical challenges need to be carefully considered when designing new studies. Recruiting and retaining sufficient numbers of participants is essential to detect statistically significant differences between groups and clearly carers need to engage with (and complete) eLearning programs in order to realize any potential benefits. More emphasis should therefore be placed on engaging and retaining study participants.

The recent published work in the area highlights that there is a tendency to neglect examining care recipient outcomes in studies evaluating the effects of online-only programs. Instead, the reviewed studies focus on measuring self-reported carer outcomes such as quality of life, stress, depression, anxiety, loneliness and care burden. Although these issues have been identified as crucial targets for supportive interventions and there is some evidence that face-to-face programs can result in improved care recipient outcomes [i.e., (34, 35)], it is currently unclear if any improvements in carers’ well-being from attending an online only program translate into better care recipient outcomes. The use of self-reported outcome measures also introduces the risk of social desirability reporting bias. Future programs and associated experimental studies should therefore consider adopting the use of subjective and objective outcome measures to evaluate both carer and care recipient outcomes.

### Nature and contents of eLearning programs

Most of the studied eLearning programs designed for the different care recipient groups included the provision of online information as one component of a more complex supportive psychoeducational intervention. The additional support varied from the provision of online group facilitators, peer-support, provision of psychological interventions from a clinician, expert health professional facilitated support, information about and links to local support organizations, technical support advisors and emailing pre-session readings in advance. More recent studies have adopted IT approaches, such as social media to promote within group communication and mobile apps. Programs with peer-support components designed to promote social connections tended to use similar communication channels, with an increasing use of social media observed in the newer studies.

As discussed previously, it is currently unclear from the existing evidence if eLearning programs without live facilitation or the provision of peer-support result in similarly effective outcomes as the complex multicomponent programs. There is a lack of controlled studies of asynchronous informal carer eLearning programs compared to inactive control groups. Therefore, evidence of their efficacy can only be estimated from studies which compare complex interventions (the experimental intervention) with asynchronous eLearning (the control intervention). For example, the recent controlled trials involving informal carers of people with mental health conditions indicate that the carers who engaged with online information resources without aspects of peer-support (i.e., asynchronous eLearning as the control condition) also experienced some improvements in outcomes. It is worth noting that in some studies, the attrition rates were higher in intervention groups with social and peer support components than in the asynchronous control groups. This suggests that informal carers could benefit from asynchronous learning and that adding social/peer support risks increasing rates of disengagement. Overall, although delivering asynchronous eLearning programs may provide an effective flexible and more accessible platform for informal carers, more evidence is required from well-controlled studies.

The extant literature provides some useful information about eLearning program contents perceived as being effective from both researchers’ and informal carers’ perspectives. Most of the evidence originates from reviews of studies designed to support informal carers of people with dementia. These reviews and some individual primary research studies indicate that effective content aimed to address both care recipient factors and carer factors. These include the prevention and management of changes in behavior associated with dementia, addressing functional deficits and how to provide practical care, such as bathing and other activities of daily living. Informal carer factors addressed in programs included improving knowledge about dementia, building carers’ identity, learning appropriate communication skills, developing positive views of the relationship with the person with dementia, caring for oneself and conceptualizing how to be a caregiver in the context of existing relationships with the care recipient. Studies also indicated that useful resources should consist of information on carers’ well-being, managing health and diseases of the care recipient, useful contacts, and technologies for eldercare. Important program contents for carers of people with mental health conditions included carers’ health literacy, CBT-related skills to improve relationships and reduce familial tension, and coping skills enhancement. Common contents observed across the limited studies including carers of adults with intellectual disabilities were basic knowledge about disabilities and common comorbidities, attachment theory, socio-emotional functioning, interpreting behaviors, responsiveness to communication signals and regulating one’s own emotions. Contents for programs designed for carers of military veterans typically encompassed self-care techniques, mental health related learning materials for new caregivers, communicating well with the medical team and effective invisible injuries treatment approaches.

The published studies often failed to report the pedagogical considerations involved in designing the programs clearly. This included intended learning outcomes, rationale and methods for formative and summative assessments and considerations about scaffolding learning. This obscures the rationale for including specific contents and complicates the replication of studies in future.

### Participant recruitment and retention

Challenges associated with recruiting, retaining and engaging sufficient numbers of informal carers were commonly reported across all care recipient groups. It seems that these issues are more evident as programs become more complex and include the provision of social/peer support. Commonly cited reasons for poor engagement and retention in the reviewed eLearning interventions also relate to the use of technology that is necessary to access the learning platforms. For example, carers frequently mention having limited IT skills and associated issues enrolling, accessing and navigating the programs. The provision of a trained facilitator, initial and clear instructions for use, technical support and emailing pre-session readings in advance seem to be useful strategies to address poor recruitment, engagement and retention. Some limited evidence also highlights that recruitment would be improved through greater investment in outreach and marketing and by reinforcing the personal relevance of attending the programs with detailed and personal marketing. Therefore, in subsequent studies more emphasis should be placed on engaging and retaining participants, which could include strategies such as offering incentives for completion, using nuanced marketing strategies, enhancing the relevance of content through co-design, offering an initial computer literacy module and the provision of ongoing technical IT support.

Table 1 summarizes our observations and recommendations for the design and evaluation of future eLearning programs for informal carers arising from this narrative review.

**Table 1:** Observations and recommendations for eLearning programs for informal carers.

Observation	Recommendations
Studies involving carers of people with dementia and those with mental health conditions indicate small to moderate improvements in different aspects of carer well-being.	Future programs/studies should include carer self-care/coping and improved communication skills as essential components to promote carer well-being. Outcomes could also consider less commonly measured aspects of well-being, such as social connections, functioning etc.
Lack of good quality research designed for informal carers of veterans and adults with disabilities.	This gap in online learning program provision and research should be addressed.
Lack of studies evaluating care recipient outcomes, therefore there is no strong evidence to show that online training leads to better care recipient outcomes.	Future program evaluations need to include measuring care recipient outcomes in addition to carer outcomes.
Most evaluations of programs are single group pre-post-test studies, uncontrolled studies, pilot/feasibility studies or include small numbers of participants.	Where appropriate, future studies should aim to control for confounding factors, include sufficient numbers of participants to provide statistical power and minimize the risk of bias by randomization of participants into groups.
Subjective outcome measures were used in most studies, which are subject to reporting bias.	Although valid and effective measures of mental well-being are used, an overreliance on subjective outcome measures risks distorting findings. Introducing an objective outcome measure of care delivery/communication (perhaps using advanced IT, such as VR care simulation) would allow assessment of caring behaviors.
There is an abundance of systematic reviews on programs for dementia carers, but a lack of good quality RCTs.	More good quality, controlled trials of eLearning programs for carers of people with dementia are required, rather than more reviews.
Unclear if fully online asynchronous programs are as effective as more complex interventions involving some live facilitation, peer-support or promotion of social connections.	Future studies should aim to ascertain the most effective components of training. This could be achieved by directly comparing different versions of a core program with no training. For example, comparing an asynchronous program with a wait list control group and a program including additional elements (i.e., peer support or live facilitated sessions) using an RCT design.
Adding synchronous professional and peer support and other intervention elements to eLearning programs may result in small-moderate benefits, but this increases the risk of dropouts.	Carefully consider (through a co-design process) the benefits and potential drawbacks of adding peer-support, social support and live facilitation elements to programs.
Lack of IT skills is a common barrier to engagement and completion of programs. Programs including the provision of IT support were appreciated by attendees and may improve engagement and retention. Unclear if older carers and those in rural settings have the required skills and infrastructure to access and complete training online.	Include IT support either via live facilitation or by including an IT introduction module as part of the training. Ensure that required IT skill levels are kept as basic as possible. Flexibility in digital platforms may encourage completion; younger carers may use apps, while older carers may prefer browsers. Several research gaps are noted, raising the following research questions: Does digital literacy exist for older populations of carers? What would be required to support this? Does infrastructure in rural/regional communities support online learning?
Many programs lack the involvement of people with lived experience in the delivery and design of training. Co-designing programs with carers and care recipients enhances relevance and may improve engagement. Similarly, people with lived experience are rarely involved in the design and conduct of research evaluating the programs.	New programs need to be co-designed and evaluated with the involvement of informal carers, the people for whom they are intended (including learning contents, program components and IT platforms). Lived experience researchers should also be engaged at an early stage of the research process.
Many studies do not report sufficient information about pedagogical approaches, intended learning outcomes and scaffolding learning. This obscures the rationale for including specific contents and complicates the faithful replication of studies.	Future studies should aim to report adequate information to allow understanding of underpinning educational rationales and study replication.
Challenges recruiting and retaining informal carer participants. There is a lack of perceived time (time-poor carers) to complete courses.	Online training advertising needs to be clear on perceived benefits to self – perhaps using personalized marketing. More research is required on how these messages effectively reach people in regional and remote communities. Co-design may enhance engagement and retention. Modules need to be quickly completed and effective training approaches adopted. Incentivization to complete training can be considered.
Practical caring skills, including supporting activities of daily living and enhancing communication, are appreciated by participants. However, it is unclear if and how these skills could be best assessed and taught.	Consider using AI and/or VR techniques to teach and assess practical caring skills where appropriate.
Informal carer self-care content is common in most programs and appreciated by participants.	Include this content for all care recipient groups.
Basic knowledge about specific conditions is included in all programs.	Relevant condition-specific information is essential. If designing a generic program for informal carers, condition-specific information could be included as optional modules (building upon several foundation modules for carers).
Carer identity is important, especially for new carers.	Include a module on developing carer identity, particularly for new carers. This should include content on promoting positive views of the relationship with the care recipient, how to be a caregiver in the context of existing relationships and balancing looking after oneself with providing care.
Social media is used increasingly, but a lack of utilizing innovative IT, such as AI and VR is noted.	Explore whether technology such as VR and AI could be developed for both teaching and assessment purposes, taking into consideration the IT literacy levels of carers.
The provision of trustworthy information and links to local support resources is viewed as helpful.	Include links to evidence-based reputable information sources and relevant local and national carer support services.

## Review limitations

The limitations of this narrative review need to be noted when interpreting the findings. This review was limited by the available data, i.e., not all training programs have been evaluated and published, so there may be excellent programs with good outcomes that were not identified. Although a quasi-systematic approach was adopted in conducting the review, we did not conduct a full systematic review as the review question was too broad. Therefore, we did not formally assess study quality, calculate effect sizes or extract data from each selected study. Studies selected for inclusion had a range of study designs and online programs were not always clearly labelled as eLearning, training or therapeutic interventions, which may have resulted in some lack of focus. For example, one study reported an evaluation of a program that was described as online learning in the published article but was defined as a series of healthcare professional-facilitated online therapeutic group sessions on the training websites, blurring the distinction between training and therapy. The current review was also conducted by one person in a relatively short timeframe and only considered articles published in the English language, potentially resulting in some relevant studies being overlooked. Finally, this review of the scholarly literature did not include input from people with lived experience, hence our interpretations of the relevance of the findings were not informed by the subjective views of carers or care recipients.

## Conclusion

Online educational and training programs for informal carers of older people, people with dementia and those with mental health conditions are likely to result in small to moderate improvements in self-reported carer well-being outcomes. The use of more complex program elements such as online peer and social support seem to be appreciated by participants, but these may increase rates of attrition and disengagement. There is currently no good quality randomized controlled trial evidence supporting the use of eLearning programs for informal carers of adults with disabilities or military veterans. There is also no clear quantitative evidence that care recipients benefit from informal carer online training programs. Future eLearning programs should be co-designed with carers and care recipients, evaluate care recipient outcomes, employ strategies to maximize recruitment and retention, support and enhance carers’ IT skills, utilize a study design that can help identify effective program components, adopt some objective outcome measures and aim to control for potential biases and confounding factors.
